# Silicone Foley catheters impregnated with microbial indole derivatives inhibit crystalline biofilm formation by *Proteus mirabilis*

**DOI:** 10.3389/fcimb.2022.1010625

**Published:** 2022-09-02

**Authors:** Mai A. Amer, Mohamed A. Ramadan, Ahmed S. Attia, Reham Wasfi

**Affiliations:** ^1^ Department of Microbiology and Immunology, Faculty of Pharmacy, October University for Modern Sciences and Arts (MSA), Giza, Egypt; ^2^ Department of Microbiology and Immunology, Faculty of Pharmacy, Cairo University, Cairo, Egypt

**Keywords:** *Proteus mirabilis*, urinary catheters, indole derivatives, crystalline biofilm, antibiofilm, gene expression

## Abstract

*Proteus mirabilis* is a common causative agent for catheter-associated urinary tract infections (CAUTI). The crystalline biofilm formation by *P. mirabilis* causes catheter encrustation and blockage leading to antibiotic treatment resistance. Thus, biofilm formation inhibition on catheters becomes a promising alternative for conventional antimicrobial-based treatment that is associated with rapid resistance development. Our previous work has demonstrated the *in vitro* antibiofilm activity of microbial indole derivatives against clinical isolates of *P. mirabilis*. Accordingly, we aim to evaluate the capacity of silicone Foley catheters (SFC) impregnated with these indole derivatives to resist biofilm formation by *P. mirabilis* both phenotypically and on the gene expression level. Silicon Foley catheter was impregnated with indole extract recovered from the supernatant of the rhizobacterium *Enterobacter* sp. Zch127 and the antibiofilm activity was determined against *P. mirabilis* (ATCC 12435) and clinical isolate P8 cultured in artificial urine. The indole extract at sub-minimum inhibitory concentration (sub-MIC=0.5X MIC) caused a reduction in biofilm formation as exhibited by a 60-70% reduction in biomass and three log_10_ in adhered bacteria. Results were confirmed by visualization by scanning electron microscope. Moreover, changes in the relative gene expression of the virulence genes confirmed the antibiofilm activity of the indole extract against *P. mirabilis*. Differential gene expression analysis showed that extract Zch127 at its sub-MIC concentration significantly down-regulated genes associated with swarming activity: *umoC*, *flhC*, *flhD*, *flhDC*, and *mrpA* (*p*< 0.001). In addition, Zch127 extract significantly down-regulated genes associated with polyamine synthesis: *speB* and *glnA* (*p*< 0.001), as well as the *luxS* gene associated with quorum sensing. Regulatory genes for capsular polysaccharide formation; *rcsB* and *rcsD* were not significantly affected by the presence of the indole derivatives. Furthermore, the impregnated catheters and the indole extract showed minimal or no cytotoxic effect against human fibroblast cell lines indicating the safety of this intervention. Thus, the indole-impregnated catheter is proposed to act as a suitable and safe strategy for reducing *P. mirabilis* CAUTIs.

## Introduction

Urinary tract infections (UTIs) account for 20% and 24% of healthcare-associated infections (HAIs) in European and developing countries, respectively, representing the major cause of this type of infections. Infections related to indwelling catheters (CAUTIs) represent 80% of HAI-UTIs (Medina and Castillo-Pino, 2019). Additionally, catheterization increases the risk of complicated infections that do not respond to treatment. *Proteus mirabilis* is a common cause of complicated UTIs associated with long-term catheterization due to the crystalline nature of their biofilm which results in catheter blockage and encrustation making the infections resistant to antibiotic treatment (Ranjbar-Omid et al., 2015).

*P. mirabilis* is an opportunistic pathogen that uses a diverse set of virulence factors to access, colonize the host urinary tract and develop a crystalline biofilm. Furthermore, the swarming motility of this bacterium facilitates their migration along the catheters (Armbruster et al., 2018). *P. mirabilis* virulence arsenal includes the flagella, fimbriae, urease enzyme, capsule polysaccharide, and efflux pumps which facilitate their colonization and survival (Wasfi et al., 2020b). Moreover, *P. mirabilis* has the ability to enter the viable but non-culturable state (VBNC) under conditions of high osmotic pressure and yet retain its virulence (Wasfi et al., 2020a).

Antimicrobial coated or impregnated urinary catheters are widely used to reduce the risk of bacteriuria, however, biofilm-induced infections associated with the use of catheters are still problematic (Pickard et al., 2012; Tenke et al., 2014). Scientists have been searching for new methods for the implementation of biofilm-free catheters. These methods include modifying catheter materials (Pathak et al., 2018; Gayani et al., 2021); coating and impregnating catheters with antimicrobial (Monteiro et al., 2019; Yassin et al., 2019) and antibiofilm compounds (Liu et al., 2019; Swidan et al., 2022). Antivirulence and antibiofilm compounds are attracting more interest recently because they affect bacterial virulence without creating selection pressure and thus reducing the development of antibiotic resistance. The role of indole as an intercellular, interspecies, and interkingdom signaling molecule has drawn the attention to this compound as a possible antivirulence compound (Lee et al., 2015). Several studies have confirmed this hypothesis by reporting the ability of some indole derivatives in inhibiting the virulence activity of many bacteria including *Escherichia coli* O157:H7, *Pseudomonas aeruginosa*, *P. mirabilis*, *Salmonella enterica* serovar Typhimurium, and *Staphylococcus aureus* (Lee and Lee, 2010; Lee et al., 2011; Lee et al., 2012a; Lee et al., 2012b; Nikaido et al., 2012; Amer et al., 2021). Thus, the present study aims to evaluate the activity of microbial indole derivatives against the formation of crystalline biofilm by *P. mirabilis* on the surface of urinary Silicone Foley catheters (SFC).

## Materials and methods

### Bacterial cultures and indole derivatives

The antibiofilm activity of the crude indole extract was tested against strong biofilm forming *P. mirabilis* isolate P8 (Amer et al., 2021), in addition to the standard strain ATCC 12453. Isolate P8 was recovered from the urine sample of a patient suffering from UTI after obtaining the participant’s consent and the approval of the Research Ethics Committee-MSA University. For the preparation of the bacterial culture, bacteria were grown in Luria-Bertani (LB) medium at 37°C overnight and the turbidity was adjusted to an optical density at wavelength 600 nm (OD_600_) 0.4 which is equivalent to 1x10^8^ CFU/mL.

Crude indole extract was prepared from a rhizobacterium *Enterobacter* sp. and coded as Zch127, followed by the identification of composing indole derivatives using LC-MS and HPLC analysis as described in our previous study (Amer et al., 2021). The extract analysis revealed the presence of 9 compounds including tryptophan, 3 methyl indole (skatole), indole-3-carboxylic acid, indole-3-acetic acid, indole-3-lactic acid, indole-3 ethanol, indole-3-pyruvic acid, indole-3 acetaldehyde, indole-3-acetonitrile, indole-3 acetamide.

Zch127 extract was selected because it showed the highest antivirulence activity against *P. mirabilis*. Stock solution of the crude indole derivative was prepared in Dimethyl sulfoxide (DMSO) at 50 mg/mL concentration for further experiments. The minimum inhibitory concentration (MIC) of crude indole extract (Zch127) was previously evaluated and reported as 1.25 mg/mL. In furtherance, sub-MIC of the extract (0.5XMIC=0.6 mg/mL) showed no significant (*p* > 0.05) inhibitory effect on the growth of *P. mirabilis* isolate (P8) and standard strain compared with the control. As an extension of our previous work, indole extract at reported sub-MIC concentration was employed for the current catheter impregnation study.

### Preparation of antibiofilm-impregnated Silicone Foley catheter (SFC)

SFC was cut into segments each of 1cm in length. Impregnation in the crude extract was done at a final concentration of 0.5X MIC (0.6 mg/mL) and kept at room temperature for 24 h (Durgadevi et al., 2019). Then, the swelled catheters were air-dried to retain their previous dimensions. Other segments were used as control which were impregnated in DMSO at the same concentration used in the test.

### Evaluation of the inhibitory effect of crude indole extract on *P. mirabilis* crystalline biofilm formation on SFC

Artificial urine was used for developing crystalline biofilm by *P. mirabilis* on impregnated SFC. The artificial urine was prepared as described by Durgadevi and co-workers (Durgadevi et al., 2020). The prepared formula was sterilized by filtration using a membrane cellulose nitrate membrane filter (0.2 µm; Sartorius, UK) after the addition of urea to a final concentration of 2.5% (w/v). The filtered artificial urine was mixed (ratio 1:1) with autoclaved double-strength LB medium.

The effect of the crude indole extract in inhibiting crystalline biofilm formation on SFC was evaluated according to the method described by Durgadevi and co-workers (Durgadevi et al., 2020) with slight modifications. Briefly, *P. mirabilis* culture was added to an artificial urine medium in a ratio of 1:100 (~1X10^6^ CFU/mL) and distributed as four ml aliquots in a 12-well cell culture plate. Each impregnated SFC catheter segment was submerged in the artificial urine medium and incubated at 37°C for 24 hours. The experiment was carried out in triplicate. After incubation, the catheter sections were gently washed with sterile physiologic buffer saline (PBS) to remove the non-adhered planktonic cells. The biofilm biomass in the control and the treated catheter was evaluated by crystal violet assay where the catheter was submerged in 1 mL of crystal violet (1% wt/vol) followed by washing with PBS and then left to air dry. The adhered stain was solubilized using absolute ethyl alcohol and measured spectrophotometrically at OD_545_ nm. The percentage of crystalline biofilm biomass inhibition was calculated using the following formula:

*% of crystalline biofilm inhibition=* $\frac{\left(Control\;OD\;545nm-Treated\;OD545nm\right)}{Control\;OD545nm}\;$ *=x100*

Viable adhering bacteria was determined according to the method described by Hou and his co-workers (Hou et al., 2020) with slight modifications. After washing, segments were sonicated by sonicator (Thermo Scientific, Waltham, MA, USA) in 2 mL PBS for 15 min followed by 5 min, followed by vortexing to detach the adherent bacteria from the catheter surface. Finally, the PBS was serially diluted (ten folds) and 10 µL were gently spread on MacConkey agar medium and incubated for 24 hours at 37°C. The reduction in adhered bacteria was calculated by the following equation:


$$log\;reduction\;in\;adhered\;viable\;bacteria=\;log_{10}\;\frac{CFUs\;on\;control\;catheter}{CFUs\;on\;treated\;catheter}$$

### Scanning electron microscope analysis of biofilms on SFC

The efficacy of antibiofilm-impregnated SFCs against *P. mirabilis* crystalline biofilm was further validated through microscopic analyses by scanning electron microscope (SEM).

The control and the antibiofilm impregnated SFC segments were submerged in artificial urine containing bacterial culture (1X10^6^CFU/mL) and incubated overnight at 37°C. After incubation, the segments were rinsed by PBS, then fixed using 2.5% glutaraldehyde followed by gradual dehydration using increasing concentrations of 20, 40, 60, 80, and 100% ethanol. The dehydrated samples were coated with gold sputter for examination under SEM at magnification power of 10,000 and 20,000x (Quanta™ 250 FEG, Thermo Fischer Scientific; New Hampshire, USA) (Yassin et al., 2019).

### Analysis of the effect of the indole extract on the swarming migration of *P. mirabilis* over urinary catheter

The anti-swarming activity of the impregnated catheters was assessed as described by Durgadevi and his co-workers (Durgadevi et al., 2020). Briefly, LB agar plates containing 1.5% agar were prepared and the agar was cut using a sterile blade into two sections. Then, segments of both the control and antibiofilm-impregnated SFCs were placed as bridges between the two sections of the agar plate. An aliquot of five microliters of bacterial culture (10^6^ CFU/mL) was inoculated into the first section of the agar plates. Then, the plates were incubated at 37°C for 24 h and observed for swarming migration of *P. mirabilis* over the catheter bridge to the other section of the plate.

### Real-time quantitative PCR(RT-qPCR) analysis of virulence gene expression

The relative change in the expression of the *P. mirabilis* (ATCC 12453) virulence genes under the effect of indole extract was studied by RT-qPCR. The change in the gene expression was assessed using bacterial culture treated with DMSO as a calibrator. Overnight bacterial culture was diluted to achieve turbidity equivalent to OD_600_ of 0.01 in LB broth supplemented with the Zch127 extract in a final concentration of 0.5X MIC (0.6 mg/mL). The bacterial cells were grown in a shaking incubator at 37°C and 250 rpm till OD_600_ of 0.8-1.0. The RNA was extracted using the RNeasy Mini Kit (QIAGEN, Germany) according to the manufacturer’s instructions. The genomic DNA was removed using the RNase-Free DNase I kit (New England Biolabs, MA, USA). The absence of the contaminating genomic DNA was confirmed by a conventional PCR reaction on the extracted RNA. The concentration and the purity of the extracted RNA were determined by a microvolume nanodrop device (Jenway, UK). One microgram of RNA was converted to cDNA using the SensiFAST™ cDNA synthesis kit (Bioline, MA, USA) according to the manufacturer’s instructions.

The effect of the indole extract on the expression level of the virulence genes was studied. These genes included; the upregulator of two-component flagellar master operon (*umoC*), flagellin transcriptional activator (*flhC*), flagellin (*flhD*), flagellin transcriptional regulator (*flhDC*), fimbrial protein (*mrpA*), MR/P fimbrial operon regulator (*mrpJ*), capsular synthesis regulator (*rcsB*), sensor kinase of the two-component flagellar master operon (*rcsD*), agmatinase (*speB*), glutamine synthetase (*glnA*) and S-ribosylhomocysteinase (*luxS*) genes involved in the biofilm formation, swarming, and amino acid metabolism. The *rpoA* gene was used as a housekeeping internal control. The real-time reaction was carried out in the Real-Time PCR System- StepOnePlus, (Applied Biosystems, MA, USA) following the reaction profile instructions supplied with the QuantiTect SYBR green master mix (QIAGEN, Germany) using the primers listed in **Table 1** and manufactured by Invitrogen (Surrey, UK). Two primers for the *mrpA* and *luxS* genes were designed for real-time PCR using the integrated DNA Technologies (IDT) primer quest tool. The specificity of all primers was tested using nucleotide Blast (Blastn) and in silico-pcr amplification (http://insilico.ehu.es/PCR/) and they showed specificity for the target genes. Each sample was analyzed in triplicates. The relative gene expression was calculated using the 2^–ΔΔCt^ method.

**Table 1:** List of primers used in quantitative PCR (qPCR).

Primer	Primer sequence (5’-3’)	Target Gene	Tm (°C)	Length	References
**rpoA_F**	GCAAATCTGGCATTGGCCCT	*rpoA*	61.6	195	(Pearson and Mobley, 2008)
**rpoA_R**	TAGGGCGCTCATCTTCTTCCG		61.9		
**umoC_F**	CACAAGCCAGCAGTACTTCA	*umoC*	58.1	144	(Aygül et al., 2019)
**umoC-R**	GTGACTCTATCGCGGCTAAA		57.5		
**flhC_F**	CGCACATCAGCCTGCAAGT	*flhC*	61.3	90	(Aygül et al., 2019)
**flhC_R**	GCAGGATTGGCGGAAAGTT		58.7		
**flhD_F**	TGCCCGTTTCTTTGTAGCAGA	*flhD*	60	148	(Pearson and Mobley, 2008)
**flhD_R**	CCGGTTTGAAGACAGCGAAA		59		
**flhDC_F**	CGCACATCAGCCTGCAAGT	*flhDC*	61.2	90	(Wang et al., 2008)
**flhDC_R**	GCAGGATTGGCGGAAAGTT		58		
**mrpA_F**	ATTGCGGGCTCTGCATTA	*mrpA*	61	113	This study
**mrpA_R**	GGAATACGTTGAGCACCTGA		61		
**mrpJ_F**	AACGTAAAGAGCTGGGTTAT	*mrpJ*	54.3	81	(Aygül et al., 2019)
**mrpJ_R**	GTTCATAGCGAGAAAACTGT		53.6		
**rcsB_F**	GCGCTTATTTGCCGAAGGTTT	*rcsB*	60.4	107	(Pearson and Mobley, 2008)
**rcsB_R**	CACCGAGCTTCATCATGGCTG		61.6		
**rcsD_F**	TCCGTATCGTACTTCCATACC	*rcsD*	56.4	86	(Aygül et al., 2019)
**rcsD_R**	GGCGAGTTTTGCAGTTATACC		57.6		
**speB_F**	GCGGGATCAAGGCAATCAAT	*speB*	68.9	126	(Aygül et al., 2019)
**speB_R**	CTTTACTGTCTTGGATGCTGC		57.2		
**glnA-F**	CGCCCAATGGTAAAAGGAGG	*glnA*	59	103	(Aygül et al., 2019)
**glnA_R**	CCACTAAGCCCATCTCTTCCA		59		
**luxS_F**	ACGATGAAAACACCCTCTGG	*luxS*	57.8	125	This study
**luxS_R**	CGCATGAAACCCGCAAATAAG		58.8		

### Cytotoxicity assay of the crude indole extract and the impregnated catheters

The cytotoxicity assay of the indole extract and the impregnated catheters was adapted from the ISO 10993-5 protocol. The assay was carried out by using healthy human fibroblast cells (ATCC CCL-75) (Hou et al., 2020; Ivanova et al., 2021).

The impregnated catheters and the control catheters were embedded in serum-free Dulbecco’s Modified Eagle Medium (DMEM) medium overnight at 37°C. Similarly, the indole extract at sub-MIC was added to the cell line medium and incubated. At the same time, the human fibroblast cell line was cultured in DMEM with 10% v/v fetal bovine serum (FBS) and 1% wt/v penicillin−streptomycin for 24 h at 37°C, 5% CO2. After achieving the confluence, 100 μL of the cell suspension with 4X10^6^ cells/mL were seeded in each well of the 96 well plate. Then, the cells were incubated in a humidified atmosphere (>90% humidity) with 5% CO2 at 37°C overnight. After decantation of the exhausted medium, 100 µL of the media interacting with the extract and catheters were pipetted to the wells in the first row of 96 well plate-containing cells (Liu et al., 2019). Media interacting with the indole extract and catheter were serially diluted (2-fold). After 24 h of incubation, the treatment medium was decanted and the cells were washed using PBS; 250 µL/well for three times. Viability of the cells was evaluated using MTT (3-[4,5-dimethylthiazol-2-yl] -2,5 diphenyl tetrazolium bromide) assay as described by Zhang and co-workers (Zhang et al., 2019) and color intensity was measured at wavelength 570nm. The percentage of cell viability was calculated using the following formula:

Viability (%)= $\;\frac{\;(OD570\;of\;tretaed\;cells)}{OD570\;of\;control\;cells\;}\;$ x100

### Statistical analysis

Statistical analysis was performed using GraphPad Prism 8.0.0 for windows (GraphPad Software Inc., CA, USA). Independent samples t-test was employed to analyse the statistical differences between extracts and DMSO-treated cultures, where *p<*0.05 was considered to be statistically significant. Two-way ANOVA analysis was used to compare the effect of the Zch127 indole extract on the gene expression levels, followed by the Bonferroni *post hoc* test to compare the replicate means, where *p<*0.05 was considered to be statistically significant. All results were presented as means ± standard deviation of three independent experiments except the reduction in adhered viable bacterial cells that was presented as median with interquartile range.

## Results

### Indole extract impregnated SFCs have a significant *in vitro* antibiofilm activity against *P. mirabilis*

**Figure 1:**
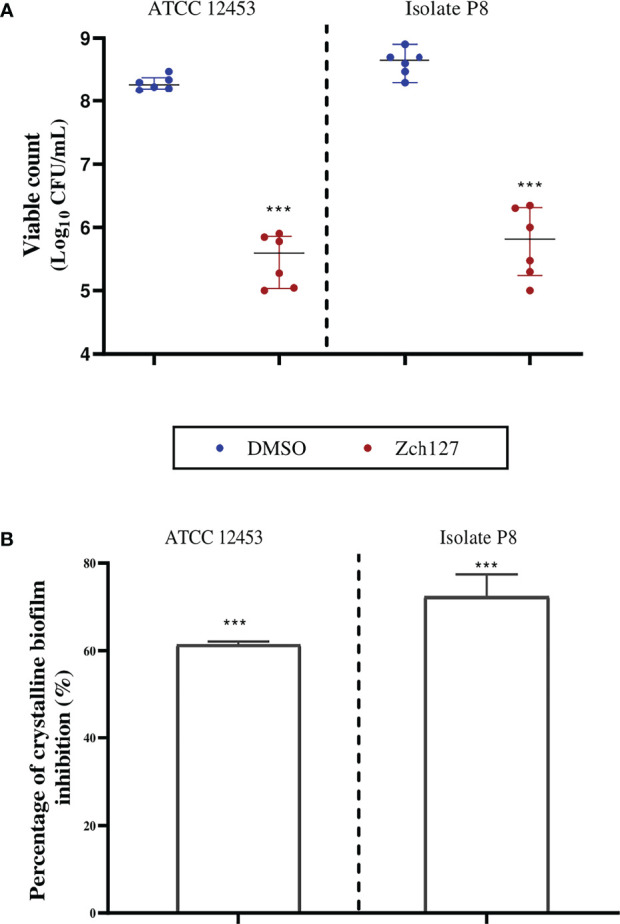
Indole impregnated silicone Foley catheter inhibit the biofilm formation by *Proteus mirabilis*. Quantification of the reduction in *P. mirabilis* crystalline biofilm on indole extract (Zch127) impregnated catheter compared to DMSO control, where **(A)** Reduction in adhered viable bacterial cells of *P. mirabilis* ATCC12453, and isolate P8 measured by drop plate method after 24 h of incubation, data are represented as median with interquartile range and **(B)** Reduction in biofilm biomass using crystal violet assay, data represents the mean of at least 3 biological replica, and error bars show standard deviation. Statistical difference was determined by student’s t-test, where statistical significance is represented by ***p < 0.001.

Catheter impregnated with the sub-MIC of the indole extract caused a significant reduction in the crystalline biofilm by three log_10_ cycles for the clinical isolate P8 and the standard strain ATCC 12453 (**Figure 1A**). The results obtained from the biofilm biomass quantification assay revealed that the impregnated SFCs caused a 60-70% reduction in crystalline biofilm (**Figure 1B**).

### SEM analysis demonstrates the superior antibiofilm activity of the impregnated SFCs

**Figure 2:**
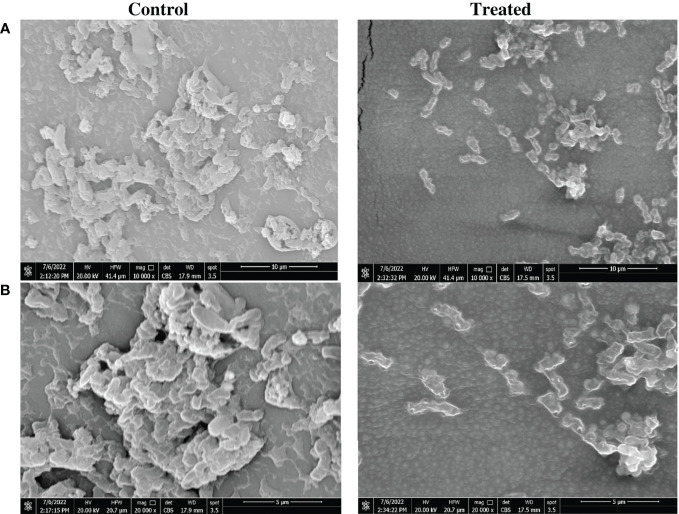
Morphological changes in *Proteus mirabilis* crystalline biofilm on indole impregnated silicone Foley catheter. Microscopic visualization of biofilm formation by *P. mirabilis* ATCC12453 on SFC segments using a scanning electron microscope (SEM). Biofilm formation on the catheter treated by impregnation at 0.5X MIC (0.6 mg/mL) of the indole extract (Zch127) was compared to control DMSO treated catheter. Visualization was done at magnifications of **(A)** 10,000 X and **(B)** 20,000 X.

Reduction of biofilm biomass on impregnated catheter was confirmed by visualization using SEM analysis. Reduction of biomass in the *P. mirabilis* biofilm on the impregnated catheter was very evident when compared to that of untreated controls. The morphology of *P. mirabilis* growing on the control catheter surface showed a high density of bacterial cells embedded in the biofilm matrix. However, indole derivative impregnated catheters displayed a clear reduction in the number of *P. mirabilis* with irregular morphology and rougher surface (**Figure 2**).

### Impregnated SFCs inhibited the migration of *P. mirabilis*

**Figure 3:**
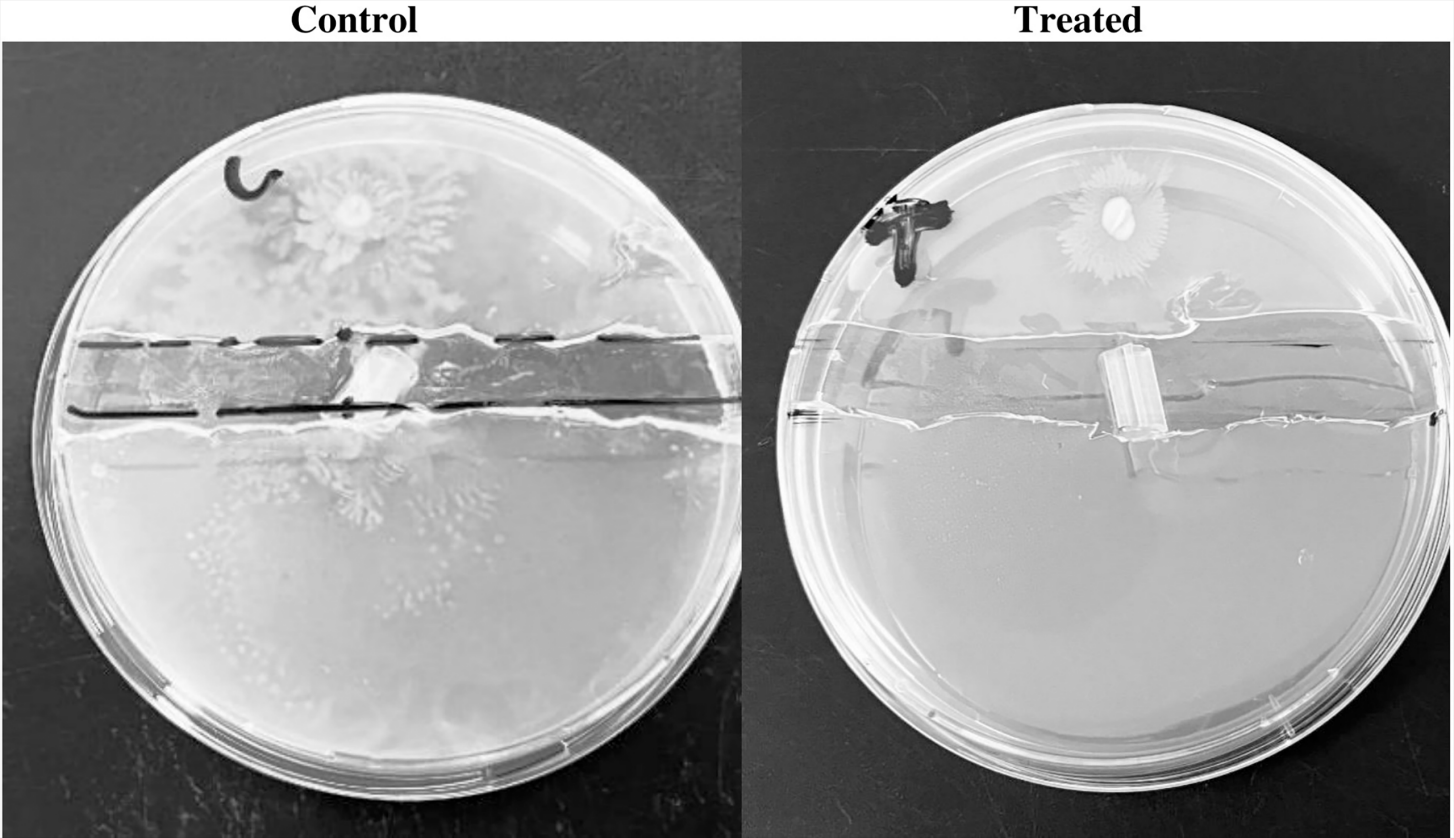
Indole impregnated silicone Foley catheter inhibit *Proteus mirabilis* migration by swarming morility. Representative images for the efficacy of impregnated SFCs against *P. mirabilis* clinical isolate P8 swarming migration, control DMSO catheter segment, and Zch127 indole extract-treated catheter segment with (0.6 mg/mL).

*P. mirabilis* swarming motility on the solid surface is crucial for the pathogenicity of *P. mirabilis in vivo*. Catheter segments impregnated with the Zch127 extract inhibited the migration of *P. mirabilis* P8 through the catheter placed in the channel between the agar sections while swarming motility was extended through the control catheter to the other agar section (**Figure 3**).

### Microbial indole derivatives downregulated *P. mirabilis* virulence genes

The RT-qPCR technique was used to evaluate the change in the gene expression in *P. mirabilis* ATCC 12453 cells upon exposure to sub-MIC of indole derivative extract (Zch127) compared to untreated control.

**Figure 4:**
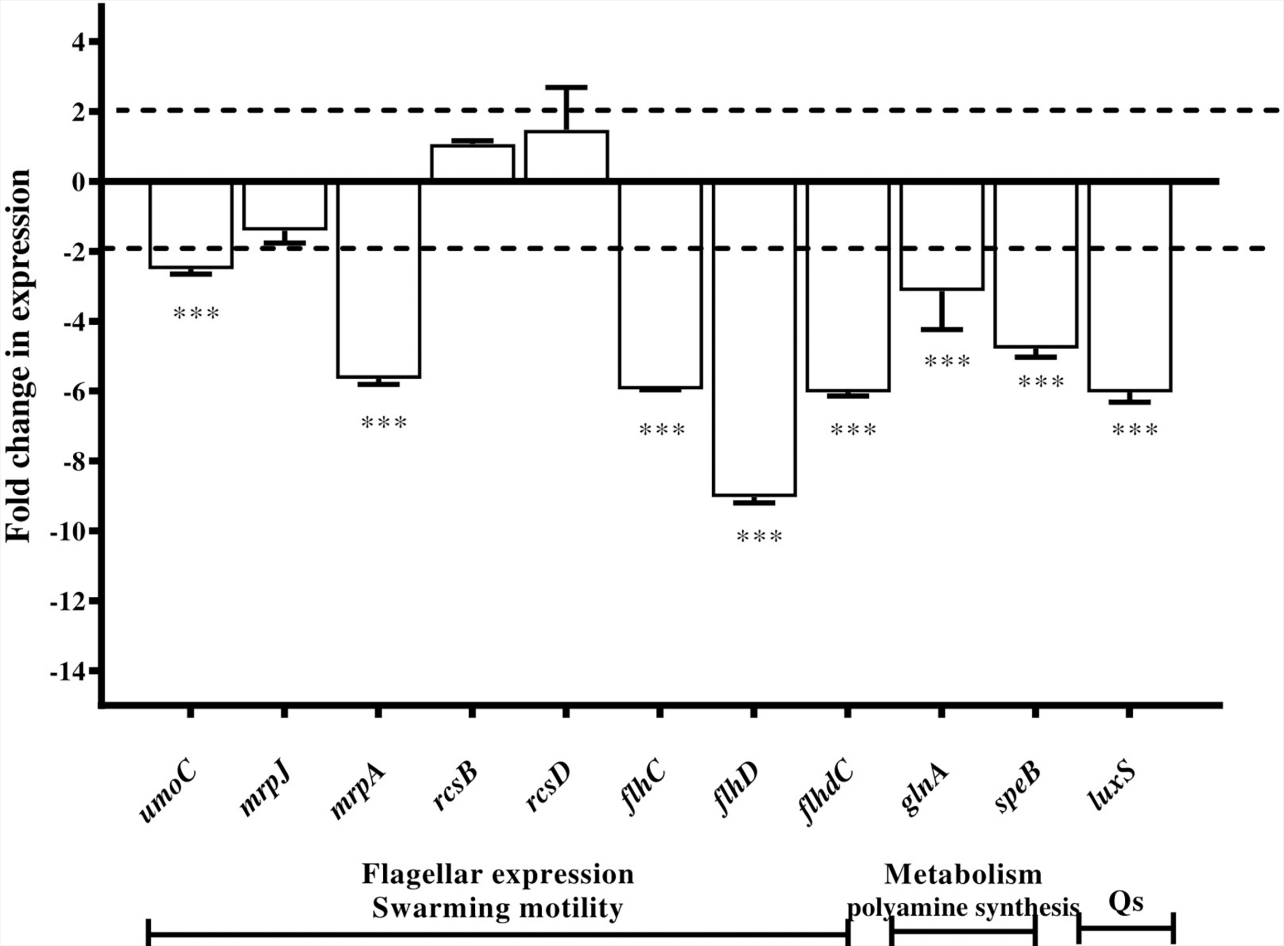
Downregulation of *Proteus mirabilis* virulence genes under the effect of indole extract. Alteration in gene expression profiles associated with exposure of *P. mirabilis* ATCC 12453 in the presence of indole extract Zch127 (sub-MIC 0.6 mg/mL) relative to control as determined by RT-qPCR.Fold change refers to the mean levels of gene expression across replicates, calculated using the ^ΔΔ^Ct method relative to the untreated control where fold change = (2 ^-ΔΔCt^). Fold change (>1) indicates up-regulation, (<1) indicates down-regulation and fold change (~1) means insignificant change. Swarming activity genes (*umoC, flhC, flhD, flhDC and mrpA*), polyamaine synthesis genes (*speB and glnA*) and quorum sensing gene (*luxS*), while regulatory genes (*mrpJ, rcsB and rcsD*). The data is representative of three independent experiments as means and error bars show standard deviation. Statistical analysis using two- way ANOVA with *p* ≤ 0.05 is considered statistically significant and ***P < 0.001 compared with DMSO control.

The level of expression of the selected genes was examined in the absence and presence of 0.6 mg/mL of extract Zch127. Expression of the selected genes was presented as a fold change in gene expression in the presence of extract Zch127 relative to the DMSO control. Differential gene expression analysis showed that extract Zch127 at its sub-MIC concentration significantly down-regulated gene associated with swarming activity (*p*< 0.001): *umoC*, *flhC*, *flhD*, *flhDC*, and *mrpA* by 2.51, 5.95, 9.03, 6.04, and 5.65 folds, respectively. In addition, Zch127 extract significantly down-regulated genes associated with polyamine synthesis *speB* and *glnA* (*p*< 0.001) by 4.78 and 3.14 folds, respectively, as well as the gene *luxS* associated with quorum sensing by 6.04 folds. Regulatory genes *mrpJ*, *rcsB*, and *rcsD* were not significantly affected by the presence of the indole derivatives (**Figure 4**)

### Absence of cytotoxic effect for indole extract

**Figure 5:**
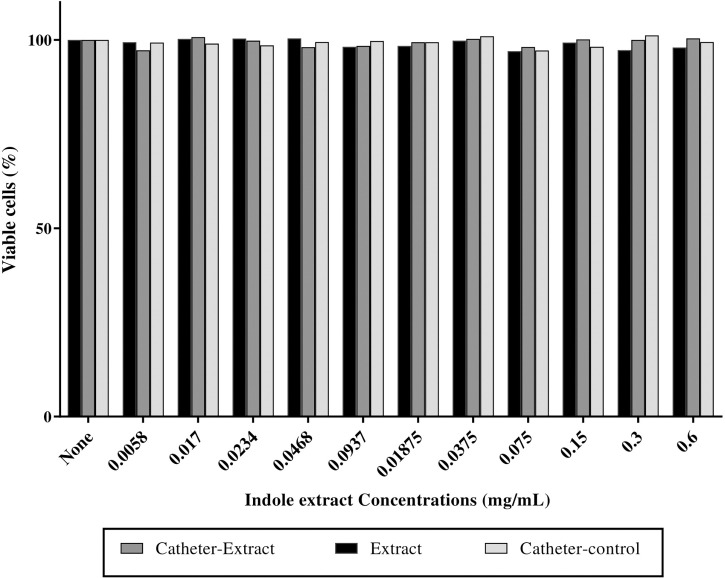
Indole extract showed no cyctotoxic effect on fibroblast cells. Cytotoxicity assay on human fibroblast cells after 24 h exposure to the nutrient medium interacting with indole extract (0.6 mg/mL), impregnated catheter with extract (0.6 mg/mL) and control catheters on human fibroblast cells. Cytotoxicity was determined by MTT assay and represented as the percentage of remaining viable cells after treatment.

No substantial change was observed in the viability of fibroblast cells at a sub-MIC concentration of impregnated catheters, and untreated catheters when compared to the untreated control cells while the extract reduced the viability of cells by 2% (**Figure 5**).

## Discussion

Catheter-associated urinary tract infections (CAUTIs) is one of the most common HAIs (Medina and Castillo-Pino, 2019). Around 150 million people are affected by these infections every year which can be complicated by kidney infections. Crystalline biofilm produced by *P. mirabilis* is a common cause of complicated and recurrent UTIs that are not responsive to regular antimicrobial treatment (Wasfi et al., 2020b). Moreover, after the removal of the catheters infected by *P. mirabilis* patient’s urine was reported (Sabbuba et al., 2003).

Treating CAUTI is difficult, therefore an alternative approach for fighting this infection could be by the prevention of biofilm formation. Over decades, catheter coating or impregnation with compounds that resist the formation of crystalline biofilm became the focus of recent studies to reduce the incidence of CAUTI (Liu et al., 2019; Gayani et al., 2021; Koc et al., 2021). Many studies suggested the utilization of antimicrobials for coating the catheters to inhibit the growth of bacteria on the surface. However, *P. mirabilis* was found to be resistant to many of these antimicrobials such as nitrofurazone and silver, thus reducing their efficiency in inhibiting *P. mirabilis-*associated CAUTI (Estores et al., 2008; Stickler, 2014). Additionally, the development of resistance to the antimicrobial coating made this therapeutic strategy less attractive. Antivirulence is an alternative approach to conventional antimicrobials for treating diseases, that focuses on interfering with bacterial virulence factors instead of the central growth pathways, thus lowering the chances of developing resistance (Dickey et al., 2017).Therefore, attenuation of the bacterial virulence factors became one of the promising therapeutic strategies against *P. mirabilis* biofilm. Recently, indole has been gaining much attention as an intercellular, interspecies, and interkingdom signaling molecule (Lee et al., 2015; Manoharan et al., 2018; Sethupathy et al., 2020), and indole derivatives have been proven to be effective against *P. mirabilis* crystalline biofilm by attenuating various virulence factors contributing to this phenotype including swarming and urease production (Amer et al., 2021). In our previous study, the indole extract Zch127 showed the highest antivirulence activity against *P. mirabilis* compared to other extracts.

At sub-MIC level, the extract reduced the swarming, urease production and biofilm formation by all tested isolates against all *P. mirabilis* isolates significantly (P<0.05) (Amer et al., 2021). In the current study, applying this extract for catheter treatment has reduced crystalline biofilm formation by 60-70% and reduced the number of adhering viable cells by three log_10_ cycles. In this study, percentage of inhibition for catheter-associated biofilm for strain P8 was similar to those detected by biofilm formation assay on microtiter plate for the same isolate in our previous study (Amer et al., 2021). This extract offers an advantage over traditional antimicrobial compounds because they do not act as an antimicrobial agent, therefore not creating stress on bacterial cells that induce resistance development, which is the problem of many antimicrobial coatings, rendering these coatings ineffective on the second and third use (Singha et al., 2017). In addition to its ability to reduce biofilm formation, this extract exhibited no cytotoxic effect on human cell line making them safe for use. In contrast, some of the antimicrobial alternatives used in coating catheters such as nanoparticles made from silver exhibt cytotoxic effect that hinders their use despite their activity against *P. mirabilis* (Akter et al., 2018; Goda et al., 2022).

**Figure 6:**
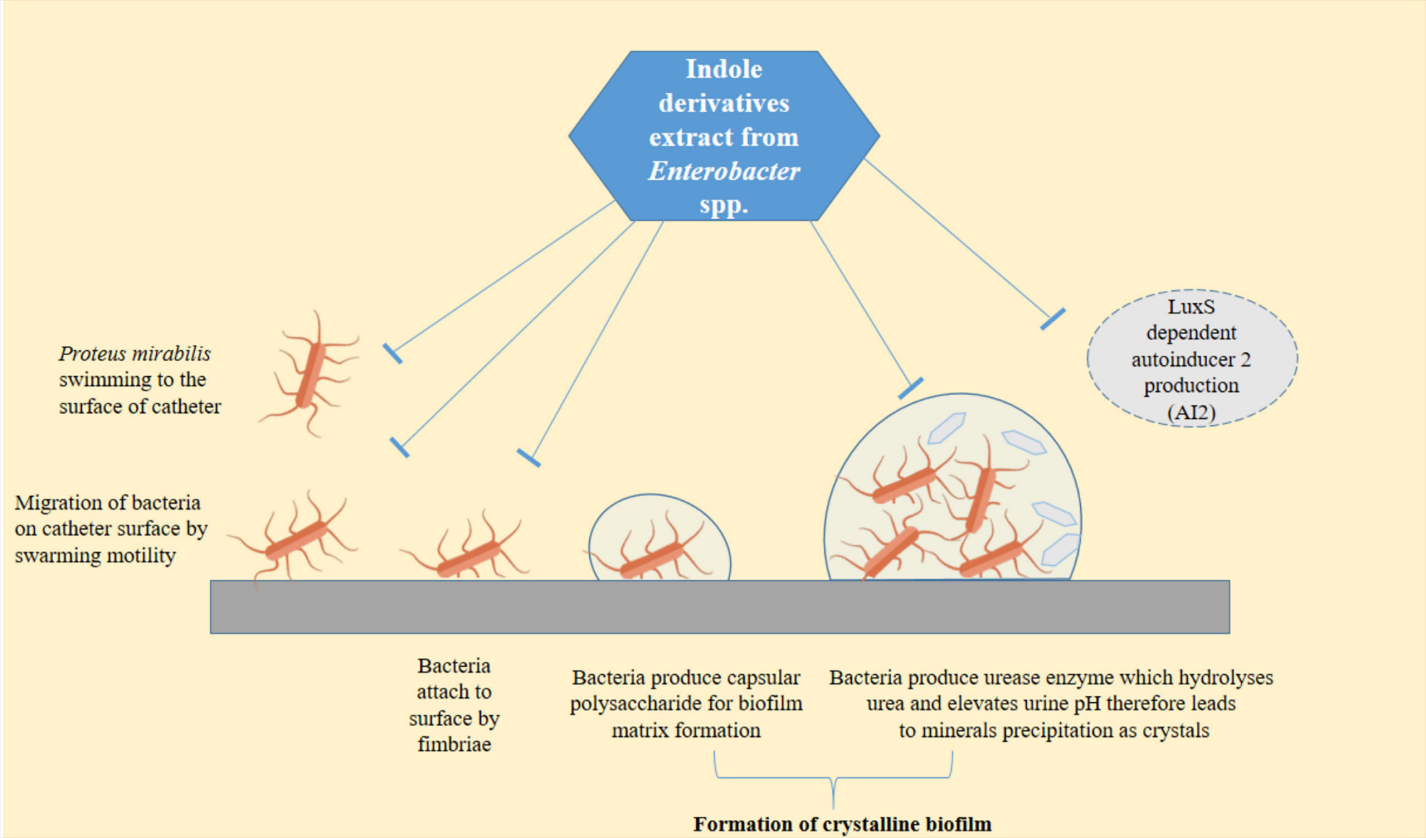
Schematic diagram summarizing steps of biofilm formation by *P. mirabilis* on urinary catheter and the effects of the indole derivative extract from the supernatant of *Enterobacter* sp. at the phenotypic and molecular levels. In this figure, lines was designed with bar-headed end indicate repression of gene expression or repression of a phenotype. Used vector of flagellated bacteria designed by Freepik (Freepik.com).

*P. mirabilis* uses a diverse set of virulence factors to access and colonize the catheter surface, including flagella, fimbriae, urease enzyme, capsule polysaccharide, and efflux pump resulting in crystalline biofilm formation. These factors allow *P. mirabilis* to attach to devices with or without the presence of a conditioning film (Wasfi et al., 2020a) **Figure 6**.

The possible mechanisms for virulence attenuation, by this extract, were studied at the gene expression level using qRT-PCR. Extract Zch127 was thought to affect the initial adherence steps of *P. mirabilis* as it downregulates significantly (*p*< 0.001) the expression of the *mrpA* gene (coding for the major subunit of the MR/K fimbriae) by 6 folds, while the *mrpJ* gene (coding for the fimbriae transcriptional regulator) was not affected. Similarly, Kim and his co-workers explained the antibiofilm effect of indole derivatives by their ability to decrease curli production (Kim et al., 2011). Another study reported that the effect of indole on biofilm formation is attributed to its role in signaling between bacterial cells at the intra and interspecies level (Lee et al., 2015). Although we did not test indole signaling in the current study but we found a significant (*p*< 0.001) reduction in the *luxS* gene (coding for AI-2 signals) expression by 6 folds, under the effect of the Zch127 crude extract which indicates interference with quorum sensing signals.

The Zch127 extract showed a unique activity against another characteristic phenotype for *P. mirabilis* which is swarming and swimming motility. Flagellar-mediated swimming motility is associated with biofilm formation because it enables the bacteria to reach the substratum and start the attachment to the surface (Pratt and Kolter, 1998) and it is under the control of quorum sensing similar to biofilm. The additional effect of Zch127 extract on *P. mirabilis* swarming and swimming motility could be a possible reason for its higher antibiofilm effect on *P. mirabilis* isolates. In our previous study, extract Zch127 inhibited P8 swarming and swimming motility up to 71% and 64%, respectively. Several studies reported that antibiofilm activity of compounds extracted from natural sources is coupled with its ability to inhibit the swarming motility (Son et al., 2010; Packiavathy et al., 2014; Marathe et al., 2018; Aygül et al., 2020). Oxidative stress plays a role in the induction and control of *P. mirabilis* swarming motility, where accumulation of reactive oxygen species (ROS), during bacterial growth, induces the cells to differentiate and migrate (Wang et al., 2006). One of the possible mechanisms for swarming inhibition is the antioxidant activity (Wang et al., 2006; Aygül et al., 2020) and that was noticed with some phytochemicals such as quercetin, resveratrol, curcumin, and epicatechin containing polyphenols with potent antioxidant activity (de la Lastra and Villegas, 2007). Similarly, some indole compounds were previously reported for their antioxidant activities in both natural and synthetic forms (Estevão et al., 2010; Juskova et al., 2011; Suzen et al., 2012; Basavaraj Shivabasappa and Jayaprakash Sharanappa, 2017; Kaya et al., 2018). Analysis of the Zch127 crude extract revealed the presence of 3 unique compounds, indole-3-acetonitrile, indole-3-carboxylic acid, and methyl indole. These compounds were reported to have high antioxidant activity by Zhao and co-workers which might explain the unique ability of the Zch127 extract to inhibit the *P. mirabilis* swarming motility (Zhao et al., 2015). Using the synthetic indole compounds for confirming the role of each derivative showed that the major contributor to the anti-swarming activity of the Zch127 was indole acetonitrile rather than indole acetic acid with swarming reduction of 85% and 14% respectively (Amer et al., 2021). The current study’s gene expression analyses confirmed the phenotypic results. Thus, it showed that the presence of extract Zch127 at its sub-MIC concentration significantly down-regulated genes associated with swarming activity (*p*< 0.001): *flhC*, *flhD*, *flhDC*, *mrpA*, and *umoC*. Moreover, the gene expression of *speB* (agmantinase) and *glnA* (glutamine synthase) were significantly down-regulated by the extract Zch127 treatment. Both genes products are important in the initiation of swarming where they participate in amino acid metabolism (Mohammad et al., 2016). On the other hand, the expression level of the swarming repressor gene *rcsD* (*rsbA*) was observed to be upregulated, which shows the negative regulation of swarming motility (Liaw et al., 2004). Thus, the qPCR data were in accordance with the results of the *in vitro* antibiofilm analysis.

Two virulence factors known to be the key players in the formation of *P. mirabilis* crystalline biofilms, the urease enzyme and the capsule polysaccharides (Jacobsen and Shirtliff, 2011). Results of our previous study revealed that the Zch127 extract caused significant a reduction in the urease activity in the *P. mirabilis* isolates in a dose-dependent manner with maximum of 96% reduction showing promising natural alternative for inhibition of crystalline biofilm (Amer et al., 2021). Whereas, capsular polysaccharide formation was not affected by the extract as revealed by qPCR results in this study. The alkaline pH, imparted by the activity of the urease enzyme, plays an essential role in the development of crystalline biofilms by *P. mirabilis*. These facts were further confirmed by the results of treating the animals with acetohydroxamic acid, a potent urease inhibitor, which reduces the severity of *P. mirabilis* infection (Musher et al., 1975; Armbruster et al., 2018).

## Conclusion

In conclusion, this study demonstrates that impregnating catheters with microbial indole extract effectively reduces crystalline biofilm formation by *P. mirabilis*. Acting on virulence factors at sub-MIC levels without affecting bacterial growth does not create much stress, therefore this reduces the probability of resistance development. Additional advantage is the safety of this compound on human cell line. Studying the change in gene expression of *P. mirabilis* in the presence of this indole extract reveals that their mechanism of action could be due to their action on the virulence factors contributing to bacterial motility, adherence, and urease production thus reducing crystalline biofilm formation by *P. mirabilis*. Further studies are intended to evaluate this approach by *in vivo* analysis *via* long-term catheterization in animal models and studying their possible synergistic activity with other antivirulence compounds to increase their activity.

## Data availability statement

The original contributions presented in the study are included in the article. Further inquiries can be directed to the corresponding authors.

## Ethics statement

The studies involving human participants were reviewed and approved by Research ethics committee, Faculty of Pharmacy, October University for Modern Sciences and Arts. The patient participant provided written informed consent to participate in this study.

## Author contributions

All authors conceptualized the work, performed the experiments, analyzed the results, edited the manuscript and contributed to the article and approved the submitted version. All authors contributed to the article and approved the submitted version.

## Conflict of interest

The authors declare that the research was conducted in the absence of any commercial or financial relationships that could be constructed as potential conflict of interest.

## Publisher’s note

All claims expressed in this article are solely those of the authors and do not necessarily represent those of their affiliated organizations, or those of the publisher, the editors and the reviewers. Any product that may be evaluated in this article, or claim that may be made by its manufacturer, is not guaranteed or endorsed by the publisher.
